# Mid-ventricular hypertrophic cardiomyopathy with apical aneurysm: a multimodality imaging case report

**DOI:** 10.47487/apcyccv.v6i1.452

**Published:** 2025-02-12

**Authors:** Pavel Martinez-Dominguez, Manuel Horna-Noriega, María José Santa-Ana-Bayona, Sara Ramírez-Flores, Lucia Horna-Regalado, Nilda Espinola-Zavaleta

**Affiliations:** 1 Departamento de Cardiología Nuclear, Instituto Nacional de Cardiología Ignacio Chávez, Ciudad de México, México. Departamento de Cardiología Nuclear Instituto Nacional de Cardiología Ignacio Chávez Ciudad de México México; 2 Facultad de Medicina y Ciencias Biomédicas, Universidad Autónoma de Chihuahua (UACH), Chihuahua, México. Universidad Autónoma de Chihuahua Facultad de Medicina y Ciencias Biomédicas Universidad Autónoma de Chihuahua (UACH) Chihuahua Mexico; 3 Departamento de Ecocardiografía, AUNA-Clínica Delgado, Lima, Perú. Departamento de Ecocardiografía AUNA-Clínica Delgado Lima Perú; 4 Departmento Cardiovascular Imaging. International Clinic, Lima, Perú. Departmento Cardiovascular Imaging International Clinic Lima Perú; 5 Universidad Peruana de Ciencias Aplicadas, Lima, Perú. Universidad Peruana de Ciencias Aplicadas Universidad Peruana de Ciencias Aplicadas Lima Peru; 6 Departamento de Ecocardiografía, Centro Médico ABC, Ciudad de México, México. Departamento de Ecocardiografía Centro Médico ABC Ciudad de México México

**Keywords:** Echocardiography, Hypertrophy, Left Ventricular, Defibrillators, Implantable, Ecocardiografía, Hipertrofia Ventricular Izquierda, Desfibriladores Implantables

## Abstract

Mid-ventricular hypertrophic cardiomyopathy is a rare subgroup within hypertrophic cardiomyopathies that may present with apical aneurysm. This condition is associated with an increased risk of cardiac adverse events, including cardiac arrest, heart failure, thromboembolic events, or sudden cardiac death. We present a case of a 41-year-old man who presented with a history of exertional dyspnea and syncope. Multimodality imaging with echocardiography and cardiac magnetic resonance showed hypertrophy of the mid-ventricular segments with apical aneurysm. An implantable cardioverter-defibrillator was implanted to prevent sudden cardiac death.

## Resumen

La miocardiopatía hipertrófica medioventricular es un subgrupo raro dentro de las miocardiopatías hipertróficas que puede presentarse con aneurisma apical. Esta condición se asocia con un mayor riesgo de eventos adversos cardíacos, incluidos paro cardíaco, insuficiencia cardíaca, eventos tromboembólicos o muerte súbita cardíaca. Presentamos el caso de un hombre de 41 años que se presentó con antecedentes de disnea de esfuerzo y síncope. La imagenología multimodal con ecocardiografía y resonancia magnética cardíaca mostró hipertrofia de los segmentos medio-ventriculares con aneurisma apical. Se implantó un desfibrilador automático implantable para prevenir la muerte súbita cardíaca.

## Introduction

Hypertrophic cardiomyopathy is an inherited disorder that affects 1 out of 500 people and increases considerably the risk of sudden cardiac death (SCD). ^1^ Mid-ventricular hypertrophic cardiomyopathy (MHC) is a rare subgroup of hypertrophic cardiomyopathy that can present with apical aneurysm. Within this subgroup, an increased risk of adverse events, including cardiac arrest, heart failure, thromboembolic events or SCD, has been reported. ^2^

The presence of hypertrophic cardiomyopathy with apical aneurysm requires a close evaluation and assessment to determine the use of an implantable cardioverter-defibrillator (ICD) aiming to prevent SCD.

We describe a patient with history of syncope, preceded by shortness of breath. Echocardiography and Cardiac magnetic resonance (CMR) demonstrated mid-ventricular hypertrophic cardiomyopathy with apical aneurysm. After evaluation and SCD risk stratification, ICD was successfully implanted. This case report emphasizes the importance of a comprehensive assessment, including clinical presentation, imaging findings, and SCD risk evaluation, in determining the appropriate management approach.

## Case report

A 41-year-old man with a 2-year history of hypertension presented with moderate exertional dyspnea and one episode of syncope. The patient reported no family history of cardiomyopathy or SCD. Upon admission, the vital signs were within normal limits, with a blood pressure of 125/72 mmHg, a heart rate of 67 beats/min, a respiratory rate of 17 breaths/min, and an oxigen saturation of 97%. Further examination revealed no significant findings.

**Figure 1:**
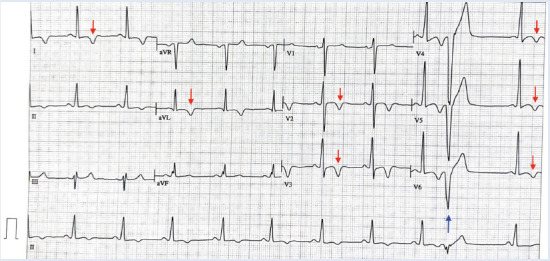
12-lead ECG revealing sinus rhythm with a heart rate of 63 beats/min. Sokolow-Lyon Criteria (S in V1 + R in V5 or V6) of 37 mm confirms left ventricular hypertrophy. Negative T waves measuring up to 5 mm in amplitude are present from V2 to V6, DI and aVL (red arrow). Premature ventricular contraction is observed (blue arrow).

An electrocardiogram (ECG) was performed, revealing sinus rhythm with 1:1 atrioventricular conduction, narrow QRS complex, and signs of left ventricular hypertrophy. Non-specific repolarization abnormalities were observed in precordial and high lateral leads, characterized by deep T wave inversions from V1 to V6 and leads I and aVL, as well as ventricular extrasystoles (Figure 1).

**Figure 2:**
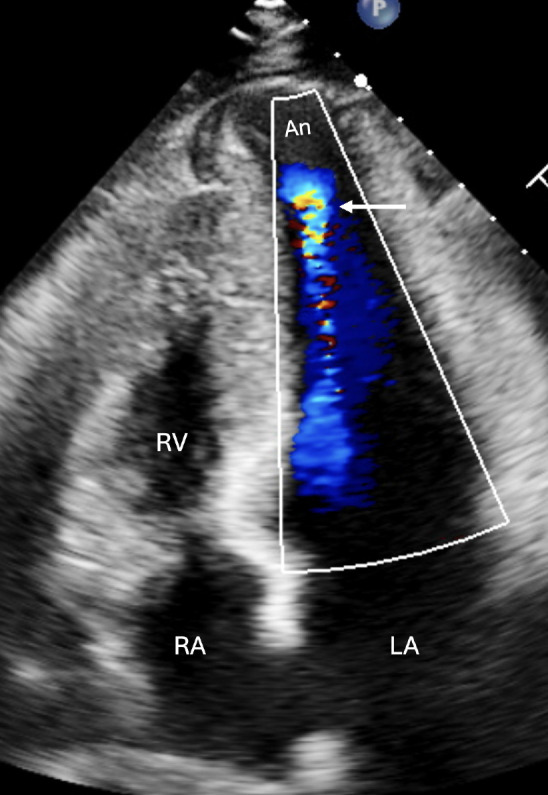
Four-chamber transthoracic echocardiographic view with color Doppler, showing an apical aneurysm (arrow). Abbreviations: An, Aneurysm; LA, Left atrium; RA, Right atrium; RV, Right ventricle.

**Figure 3:**
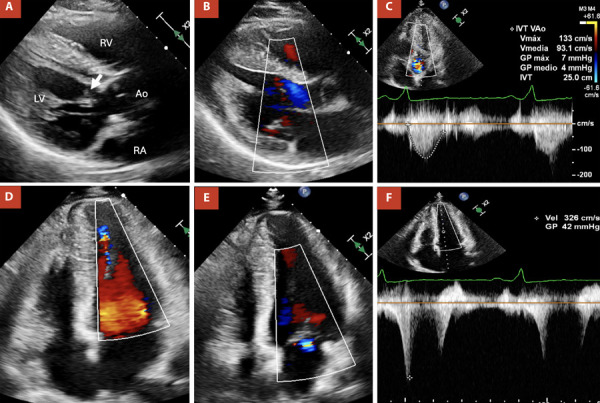
A) Parasternal long-axis view showing redundant chordae tendineae (arrow). B) Parasternal long-axis view with color Doppler, demonstrating the absence of subaortic obstruction (Laminar flow). C) Apical five-chamber view with color and continuous-wave Doppler, showing no evidence of subaortic obstruction. D) Four-chamber view with color Doppler, revealing turbulence in the apical portion of the left ventricle. E) Four-chamber view showing mild mitral regurgitation. F) Four-chamber view with color and continuous-wave Doppler demonstrating apical obstruction with a maximum gradient of 42 mmHg, partially contaminated by mitral regurgitation flow.

Subsequent diagnostic assessment included a transthoracic echocardiogram (TTE). (Figures 2 and 3; Videos 1 and 2) This study reported a preserved left ventricle ejection fraction of 68%. However, there was evidence of hypertrophy in the middle-to-apical segments of the left ventricle, with a maximum wall thickness of 20 mm and a midventricular gradient of 42 mmHg. It also demonstrated signs of apical aneurysm with dyskinesia. Additionally, mild left atrial dilation (36 mL/m2), grade II diastolic dysfunction (E/A = 1.5, E’ = 4 cm/s), increased end-diastolic pressure (E/E’ = 15), and mild mitral regurgitation were observed.

**Figure 4:**
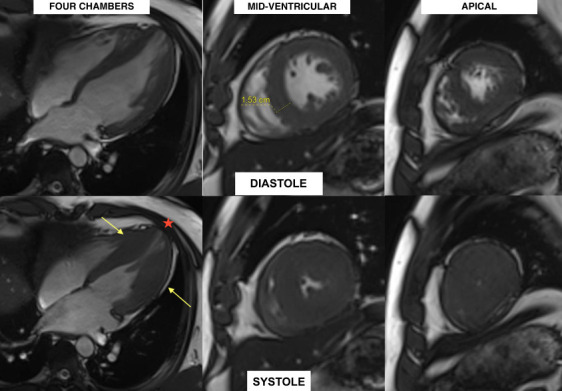
Cardiac magnetic resonance with four-chamber, mid-ventricular and apical views during diastole (above) and systole (below) showing mid-ventricular hypertrophy of 15.3 mm (yellow arrows) with apical aneurysm (red asterisk). Increased trabeculations can be seen in anterolateral, basal, medial, and lateral apical segments.

**Figure 5:**
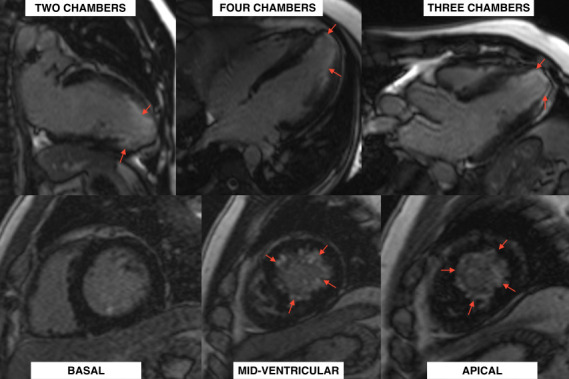
Late gadolinium enhancement resonance with two, three, and four-chamber views (above) and basal to apical views (below) showing subendocardial late gadolinium enhancement in mid-apical segments less than 15% of the total ventricular mass (red arrows).

A CMR was performed, which confirmed mid-ventricular hypertrophy of up to 15 mm in the infero-septal medial segment, along with the formation of an apical aneurysm (Figure 4). Late gadolinium enhancement (LGE) revealed patchy myocardial fibrosis in the middle and apical segments, accounting for less than 15% of the total left ventricular mass (Figure 5). No edema was detected in the T2 STIR and T2 mapping sequences.

In addition, during an exercise stress test, a normal blood pressure response was observed, and no arrhythmic events were demonstrated in the 24-hour Holter monitoring. According to the 2020 AHA/ACC Guideline for the Diagnosis and Treatment of Patients With Hypertrophic Cardiomyopathy, ^3^ the patient had a 5.22% of SCD in the following 5 years. Considering the SCD-risk and imaging findings, the patient had a class 2A recommendation for the implantation of a cardioverter-defibrillator (ICD) for primary prevention, which was successfully implanted. 

## Discussion

Multimodal imaging is crucial in differentiating the etiology of myocardial hypertrophy, particularly in distinguishing hypertrophic cardiomyopathy from hypertensive heart disease and other infiltrative or metabolic disorders. ^4^^)^ Echocardiography remains the first-line modality, with characteristic findings varying by etiology. Hypertrophic cardiomyopathy typically presents with asymmetric septal hypertrophy, which can lead to dynamic left ventricular outflow tract obstruction (LVOT), often accompanied by systolic anterior motion (SAM) of the mitral valve.^5^ In contrast, hypertensive heart disease usually exhibits concentric left ventricular hypertrophy without LVOT obstruction and with preserved global longitudinal strain until later disease stages. ^6^

CMR provides superior myocardial characterization. LGE helps distinguish hypertrophic cardiomyopathy, which often shows patchy fibrosis at the right ventricular insertion points and in hypertrophied segments, from hypertensive heart disease, where fibrosis is more diffuse and subendocardial. Apical HCM, often underdiagnosed on echocardiography, is best visualized on CMR. ^7^ Meanwhile, amyloidosis is characterized by global LV thickening, diffuse subendocardial LGE, and markedly elevated native T1 and extracellular volume fraction. ^8^

MHC is a rare and infrequent form of hypertrophic cardiomyopathy, frequently associated with apical aneurysms. Apical aneurysms are present in less than 5% of patients with hypertrophic cardiomyopathy; however, they are more common in those with the mid-ventricular subtype. ^9^

The mechanism described for apical aneurysm formation is unclear. One mechanism suggests that an increased gradient between the basal and apical segments of the ventricle leads to elevated apical pressure and myocardial stress, culminating in wall weakening and formation of the aneurysm through a chronic and dynamic process.^9^

In patients with hypertrophic cardiomyopathy, echocardiographic criteria include a left ventricular thickness above 15 mm or above 13 mm if the patient has a first-degree relative with hypertrophic cardiomyopathy, in the absence of other causes of hypertrophy. Other signs of hypertrophic cardiomyopathy include mitral valve disease, left ventricle diastolic dysfunction, or mild atrial enlargement. ^10^

In our patient, the presence of these signs confirmed the important role of echocardiography in the assessment and diagnosis of MHC. Further examination with CMR allows to identify and measure the wall thickness, obstruction, and confirm the presence of aneurysms. ^11^ Moreover, CMR modalities such as late gadolinium enhancement, STIR, T2 are part of the routine evaluation in hypertrophic cardiomyopathy. ^12^

Patients with hypertrophic cardiomyopathy and late gadolinium enhancement are more likely to show a decreased ejection fraction and increased major adverse cardiac events.^13^ Thus, MHC represents a high-risk subgroup of hypertrophic cardiomyopathy, as reports have shown increased risk of ventricular arrhythmias such as nonsustained ventricular tachycardia (NSVT), sudden cardiac death, thromboembolic events, and heart failure. ^2^ NSVT mechanism is not well understood; however, it has a reported prevalence of 20%-30%.^14^ Additionally, there is an 8-fold increase in SCD in patients with hypertrophic cardiomyopathy accompanied by aneurysm than without aneurysms. ^11^

Initial treatment includes pharmacological therapy with beta-blockers and antiarrhythmics to prevent arrhythmias. ^15^^)^ Alternatives such as alcohol septal ablation, cryoablation and surgical myectomy may also be considered in patients with refractory symptoms despite medications. ^16^ Even though these interventions have shown good outcomes, further studies are needed to determine the appropriate timing and patient selection for these procedures. ^17^

Assessment of SCD is crucial during hypertrophic cardiomyopathy evaluation, as there are established risk factors for SCD, including age, family history, recent unexplained syncope, history of NSVT and echocardiographic parameters such as left atrial size, ventricular thickness, ventricular gradient.^1^ The 2020 AHA/ACC guidelines include left ventricular systolic dysfunction, apical aneurysm, and extensive LGE on CMR as risk factors for SCD. Risk stratification allows to determine if an ICD is necessary for primary prevention. ^3^

There is a difference between the current European and American guidelines regarding the role of aneurysm in sudden cardiac death. The ACC/AHA consider apical aneurysms as a risk factor for SCD and suggests ICD implantation in these patients, while the ESC guidelines suggest there is insufficient data and only consider apical aneurysm as class IIa for indication of ICD implantation. ^4^ However, after comprehensive evaluation of this patient, the cardiovascular team decided to implant an ICD to prevent future cardiac complications. This decision was supported by the presence of an apical aneurysm and the history of syncope.

In conclusion: MHC is a rare and high-risk subgroup of hypertrophic cardiomyopathies. The presence of apical aneurysm in these patients requires a comprehensive assessment including SCD-risk stratification to consider ICD implantation. We aim to underscore the importance of imaging in order to have a timely intervention and elucidate the significant role that ICDs play in SCD prevention within this unique subgroup, however further studies to understand how to appropriately manage these patients are needed.
